# Distribution and frequency of G119S mutation in *ace*-*1* gene within *Anopheles sinensis* populations from Guangxi, China

**DOI:** 10.1186/s12936-015-1000-0

**Published:** 2015-11-25

**Authors:** Xiangyang Feng, Chan Yang, Yichao Yang, Jun Li, Kangming Lin, Mei Li, Xinghui Qiu

**Affiliations:** Guangxi Zhuang Autonomous Region Centre for Diseases Control and Prevention, Nanning, 530028 China; State Key Laboratory of Integrated Management of Pest Insects and Rodents, Institute of Zoology, Chinese Academy of Sciences, Beijing, 100101 China; University of Chinese Academy of Sciences, Beijing, 100049 China

**Keywords:** Acetyl-cholinesterase, *Anopheles sinensis*, G119S mutation, Guangxi Zhuang Autonomous Region of China, Organophosphate, Carbamate, Insecticide resistance

## Abstract

**Background:**

Malaria is one of the most serious vector-borne diseases in the world. Vector control is an important measure for malaria prevention and elimination. However, this strategy is under threat as disease vectors are developing resistance to insecticides. Therefore, it is important to monitor mechanisms responsible for insecticide resistance. In this study, the presence of G119S mutation in the acetyl cholinesterase-encoding gene (*ace*-*1*) was investigated in nine *Anopheles sinensis* populations sampled across Guangxi Zhuang Autonomous Region China.

**Methods:**

PCR–RFLP (polymerase chain reaction-restriction fragment length polymorphism) method was used to genotype each individual adult of *An. sinensis*. Direct sequencing of PCR products was performed to verify the accuracy of PCR–RFLP genotyping result. Population genetics analysis was conducted using Genepop programme.

**Results:**

The frequencies of susceptible homozygotes, heterozygotes and resistant homozygotes in the nine populations ranged between 0–0.296, 0.143–0.500 and 0.333–0.857, respectively. Overall, a high frequency (0.519–0.929) of mutant 119S allele was observed and the genotype frequency of the *ace*-*1* gene of *An. sinensis* was at Hardy–Weinberg equilibrium in each of the nine examined populations.

**Conclusion:**

The G119S mutation has become fixed and is widespread in *An. sinensis* field populations in Guangxi, China. These findings are useful in helping design strategies for *An. sinensis* control.

## Background

Malaria is one of the most serious vector-borne diseases, representing a major threat to global public health [1]. Vector control has been proven to be an important component in malaria prevention and elimination programmes. The use of different classes of insecticides has played an essential role in controlling mosquitoes, but also has resulted in the development of insecticide resistance [1, 2]. Insecticide resistance is well regarded as a major obstacle in vector control, thus resistance monitoring is critical for establishing smart vector management strategies [1].

Organophosphates (OP) and carbamates (CM) have been used for agriculturally important pest and disease-vector control. The primary molecular target of OP and CM is the acetylcholinesterase (AchE, EC 3.1.1.7). Inhibition of insect AchE leads to the accumulation of acetylcholine, thus terminates nerve impulses in cholinergic synapses and eventually causes death [3]. Previous studies have demonstrated that point mutations in AchE are associated with insecticide resistance against OP and CM [3]. For example, a point mutation leading to a single amino acid substitution of glycine to serine at position 119 (G119S, *Torpedo californica* numbering) in the AchE1 [4], is associated with OP and CM resistance in several important mosquito species [4–16]. This resistance-associated mutation provides a molecular marker for detecting or monitoring CM and OP resistance in these mosquitoes.

Guangxi Zhuang Autonomous Region was once a malaria-endemic region. Since the launch of the National Malaria Control Programme in 1955 in China, the malaria morbidity rate in Guangxi reduced from 296.67 per 10,000 people in 1954 to below 1 per 100,000 people during 2000 to 2011 [17]. Although indigenous malaria has been basically eliminated in Guangxi, many imported cases of malaria have been identified in returning workers from Africa and southern Asia [17]. In addition, increased population migration (especially cross-border migration) and the possible change of vectorial capacity may enhance the risk of malaria re-emergence [18]. This situation underlines the necessity of malaria prevention through effective vector control.

**Fig. 1:**
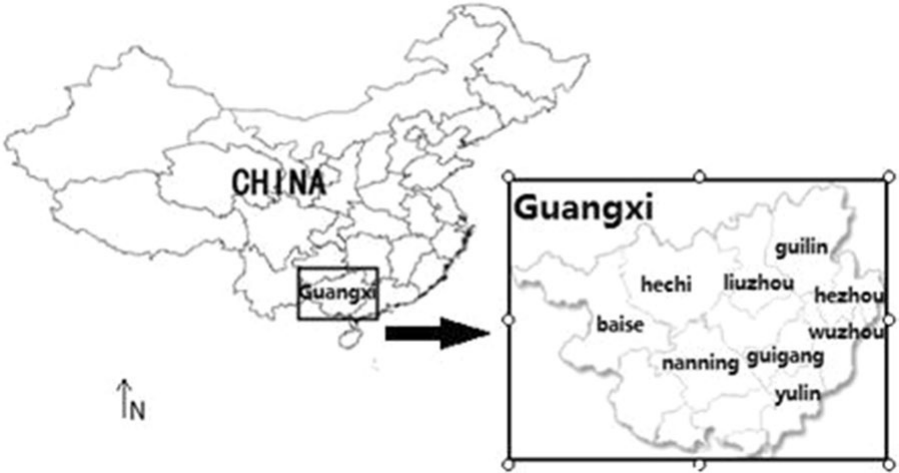
Sampling sites of *Anopheles sinensis* in Guangxi, China

In Guangxi, the major vector for transmitting malaria is *Anopheles sinensis* [17]. Guangxi is located in the southern part of China (Fig. 1), where there are a lot of rice fields providing a sound environment for *An. sinensis* breeding. Wild *An. sinensis* populations continue to be exposed to various insecticides used in surrounding rice paddies, thus insecticide resistance is expected to be selected. However, until recently, the status of insecticide resistance and its associated genetic mutations in *An. sinensis* field populations in Guangxi is less understood. Therefore, there is an urgent need to detect possible insecticide resistance in *An. sinensis* populations to avoid failure in the effort of vector control. In this study, evidence on the presence and the frequency of the G119S mutation of *ace*-*1* gene conferring organophosphate (OP) and carbamate (CM) resistance in Guangxi was provided.

## Methods

### *Anopheles sinensis* collection

*Anopheles sinensis* adults used in the study were caught by light (wave length 365 nm) trap from July to September in 2014, around farmers’ houses in Nanning, Yulin, Hezhou, Baise, Wuzhou, Liuzhou, Guilin, Hechi, and Guigang of Guangxi (Fig. 1). Individual mosquitoes were morphologically identified and the confirmed *An. sinensis* adults with a high level of confidence were put into 100-μl Eppendorf tubes containing 100 % ethanol solution, and kept at 4 °C until use. Ten randomly selected adults from each population were further identified using the rDNA-ITS2 method [19]. The identities of the molecularly identified specimens matched perfectly to their morphological identifications.

### Genomic DNA extraction

Genomic DNA of individual mosquitoes was prepared according to the method of Rinkevich [20]. Genomic DNA samples were stored at −20 °C.

### *Ace*-*1* genotyping

PCR–RFLP was conducted for genotyping the *ace*-*1* gene at codon 119. Primers As-ace-F and As-ace-R [6] (commercially synthesized by Invitrogen, China Service) were used to amplify a fragment encompassing codon 119 of the *ace*-*1* gene of *An. sinensis*. Reaction system contained 10 × Buffer 3 μl, dNTP 3 μl, rTaq DNA polymerase (Takara) 0.3 μl, DNA template 5 μl, As-ace-F 0.6 μl, As-ace-R 0.6 μl, ddH_2_O 17.5 μl. PCR parameters were set as 95 °C for 5 min, 36 cycles of 95 °C for 30 s, 52 °C for 30 s and 72 °C for 40 s, followed by 72 °C for 10 min and 4 °C forever. PCR products were detected on a 1.2 % agarose gel.

Restriction endonuclease AluI (New England Biolabs) was used for genotyping. This enzyme can cut the 119S-type (mutant) PCR product into two bands, but cannot cut the G119-type (wild) PCR product due to its specific recognition site (AGCT). The digestion reaction consisted of PCR product 10 μl, Cutsmart buffer 2 μl, AluI (10 unit/L) 0.4 μl, ddH_2_O 8 μl in a total volume of 20 μl. After reaction for 4 h at 37C°, the digestion products were detected on a 1.2 % agarose gel. Direct sequencing was performed for confirming the reliability of PCR–RFLP in genotyping by GBI tech (Beijing Service, China).

### Data analysis

Bioinformatics analysis was conducted using online programmes. Population genetic parameters were calculated using Genepop 3.4, and Chi-test was performed by SAS 9.2.

## Results

**Fig. 2:**
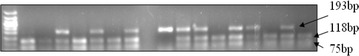
Electrophoresis detection of restriction endonuclease digestion product. The first lane is DNA marker, and the other lanes represent individual fly samples. The lanes showing two DNA bands (118 and 75 bp) define resistant homozygotes (SS); the lanes showing three bands (193, 118 and 75 bp) define the hyterozygotes (GS); the lanes showing single band (193 bp) defines susceptible homozygotes (GG)

**Fig. 3:**
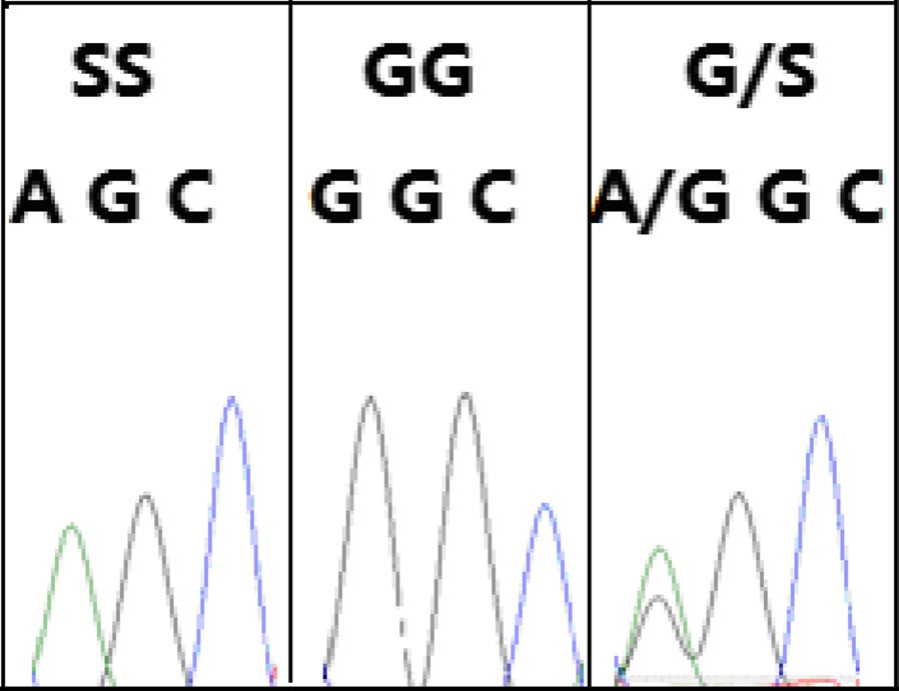
The example of nucleotide sequence chromatograms (codon 119 of *ace*-*1* gene) of three genotypes identified in *Anopheles sinensis.* At the first locus, the resistant homozygote (SS) has a single nucleotide (A) peak, the susceptible homozygote (GG) has a single nucleotide (G) peak, and the heterozygote (G/S) has double nucleotide (A and G) peaks

All the possible three genotypes were identified in a total of 312 individuals sampled from nine locations across Guangxi (Fig. 1), i.e., wild-type (susceptible) homozygote GG, heterozygote GS and mutant (resistant) homozygote SS, which corresponded to one band (193 bp), three bands (193 bp + 118 bp + 75 bp) and two bands (118 bp + 75 bp) in the PCR–RFLP profile, respectively (Fig. 2). The accuracy of genotype was confirmed by direct DNA sequencing of corresponding purified PCR products, i.e., the expected mutation (G to A) at the first locus of codon 119 was identified in heterozygotes (GS) and resistant homozygotes (SS) (Fig. 3).

Wild-type homozygote had a low frequency that ranged between 0 and 0.296 (Table 1). Notably, no wild-type homozygote was detected in the samples from five locations (Nanning, Hezhou, Wuzhou, Hechi, and Guigang). The frequencies of heterozygotes and resistant homozygotes ranged between 0.143–0.500 and 0.333–0.857, respectively. Overall, high frequencies (0.519–0.929) of the mutant allele were observed in all the nine enrolled populations (Table 1).

**Table 1:** Allele frequency of *ace*-*1* in *Anopheles sinensis* populations sampled across Guangxi, China

Locations	Size	SS	GS	GG	119S frequency	Genotype χ^2^-test (*p* value)	Heterozygote excess (*p*-value)	Heterozygote deficiency (*p*-value)
Nanning	36	23	13	0	0.819	0.450	0.268	1.000
Yulin	52	25	23	4	0.702	0.943	0.499	0.750
Hezhou	19	12	7	0	0.816	0.666	0.514	1.000
Baise	56	35	17	4	0.777	0.602	0.917	0.258
Wuzhou	26	22	4	0	0.923	0.936	0.880	1.000
Liuzhou	27	9	10	8	0.519	0.355	0.967	0.148
Guilin	32	14	16	2	0.688	0.702	0.338	0.901
Hechi	49	42	7	0	0.929	0.878	0.790	1.000
Guigang	15	9	6	0	0.800	0.684	0.542	1.000

No heterozygote excess or heterozygote deficiency was observed in all the nine populations (Table 1). Chi-test indicated that there was no significant difference between observed number and expected number of individuals of each genotype in each population (Table 1). These parameters suggested that all the nine populations of *An. sinensis* in Guangxi were at Hardy–Weinberg equilibrium (HWE).

## Discussion

Genotyping results reveal that there was a high frequency of 119S resistance allele in each of the nine field populations of *An. sinensis* collected across Guangxi (Table 1). Notably, the average frequency of resistance allele was close to 0.8, and an even higher mutation frequency (higher than 0.9) was observed in Wuzhou and Hechi populations. These observations clearly demonstrate that the G119S mutation is prevalent throughout Guangxi. Also in China, modest to high (0.45–0.75) frequency of resistance allele was observed in *An*. *sinensis* populations from Hainan Island [16], and Yunnan (0.385) and Anhui (0.589) provinces [6]. Similar to results obtained in this study, the frequency of the 119S (mutant) allele was determined to range from 0.744 to 0.972 in ten local field populations of *An*. *sinensis* in Korea [15]. These results indicate that the G119S mutation is widely distributed in Asia.

**Fig. 4:**
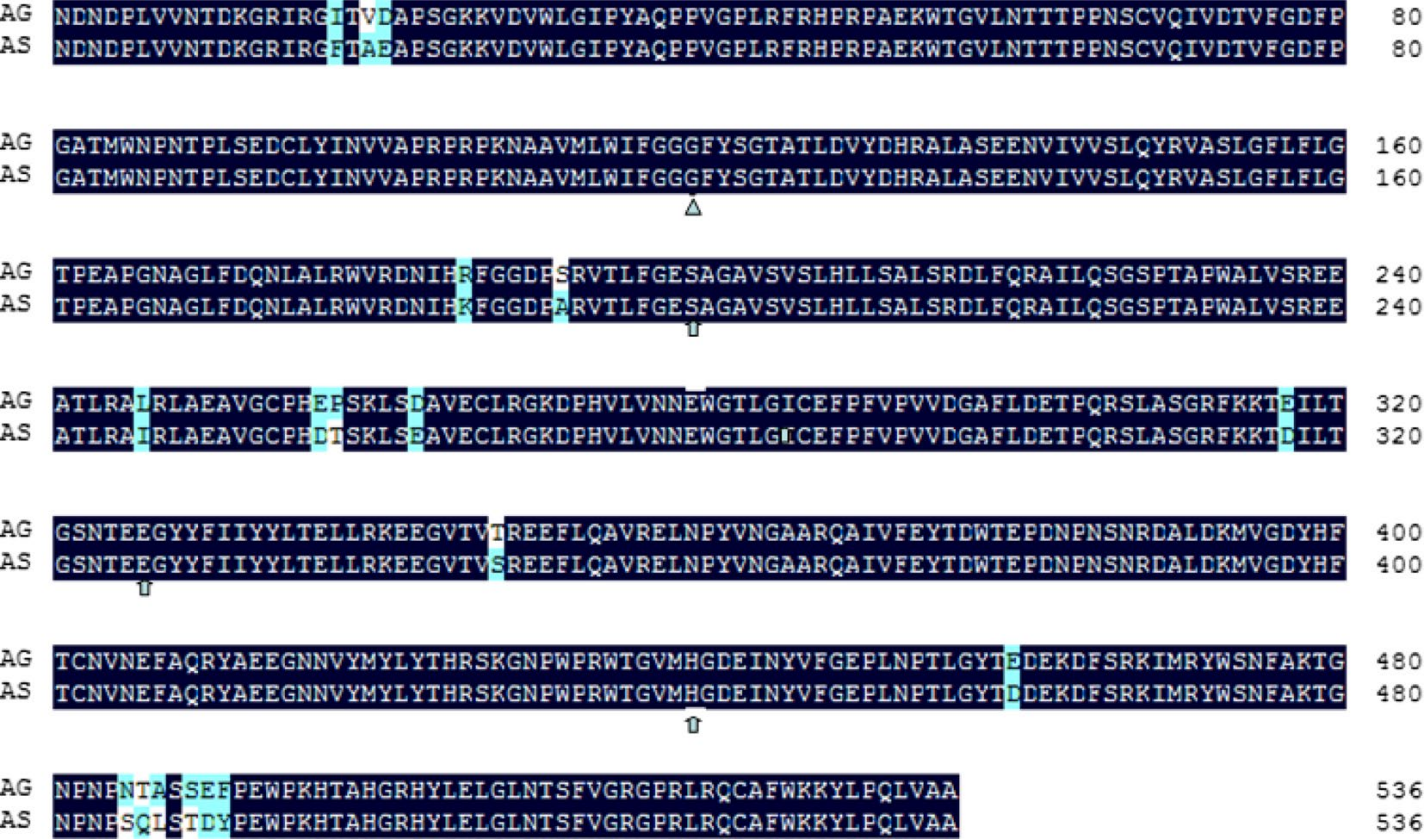
Alignment of AChE1 mature protein sequence of susceptible *An. gambiae* (AG, Kisumu [23]) and *An. sinensis* (AS, this study). AS sequence was obtained by directly sequencing PCR product using cDNA templates prepared from RNA of ten adults of a susceptible strain of *An. sinensis* [25], and the PCR primers were designed based on the whole genome shotgun sequences (KE524393 and KE524938).The Glycine 119 residue is marked with a triangle. The three residues (S200, E327, H440) forming the catalytic triad were marked with arrows

The amino acid substitution of glycine with serine at position 119 (G119S) is able to reduce the sensitivity of AchE1 to OP and CM [3]. Previous studies have elucidated that the G119S mutation of the *ace*-*1* gene in *Anopheles* and *Culex* mosquitoes is associated with insect resistance against OP and CM [4–16]. For example, the Ace-1^R^ allele is strongly associated with survival of *An. gambiae* mosquitoes from Côte d’Ivoire after exposure to bendiocarb and fenitrothion [21], and G119S in *An. gambiae* from Accra (Ghana) is strongly associated with resistance [10]. In addition, it is known that the strength of resistance is expressed in a partially dominant manner in *An. gambiae* [22]. Sequence alignment (Fig. 4) reveals a very high identity of amino acid sequence between the deduced *An. sinensis* AchE1 and *An. gambiae* AchE1 (96.6 % in the total 536 amino acids of the mature protein), and no difference was observed in amino acids determining catalytic function of cholinesterase [23]. It is logical to think that AchE1 in these two species has similar biochemical properties, and the conserved G119S mutation will confer similar resistance profiles to OP and CM [23]. The high frequency of the resistance allele and high ratio of mutant homozygotes in all the tested *An. sinensis* populations strongly suggest that G119S resistance mechanism against OP and CM is widespread in Guangxi, hence vector control strategies based on these two classes of insecticides may not be effective as a consequence. However, given that the resistance level may largely vary depending on insecticides even for a given resistance mechanism [22], the relationship between the frequency of mutant allele and the strength of resistance to specific insecticides in these populations could not be exactly established because the susceptibility data is not available for the samples used in this study. In addition, considering that mosquitoes can express multiple insecticide-resistance mechanisms in the field [10, 21], other factors such as over-expression of metabolic genes may be attributable to OP and CM resistance in *An. sinensis* populations of Guangxi. Further studies are required to clarify a causal role for G119S mutation in *ace*-*1* in OP and CM resistance, and to characterize other possible involved mechanisms.

In contrast to the study in West Africa observing a significant departure from Hardy–Weinberg equilibrium (HWE) in some field samples of *An. gambiae* [24], population genetics analysis shows that the genotype frequency at codon 119 of the *ace*-*1* gene was at HWE in all the nine examined populations of *An. sinensis* from Guangxi, with no heterozygote excess or heterozygote deficiency being observed (Table 1). These parameters indicate that mosquitoes carrying G119S mutation may suffer no fitness cost under current natural conditions. If this is the case, actions to eliminate resistant individuals and limit the spread of the resistant population should be taken. Whether G119S genotype expresses fitness cost in *An. sinensis* remains to be characterized.

## Conclusions

This study demonstrates that G119S mutation has become fixed, and is widespread in *An. sinensis* field populations across Guangxi, China. The high frequency of G119S mutation and high ratio of mutant homozygotes may allow these mosquito populations to be resistant to OP and CM. These findings emphasize the need to monitor insecticide resistance and to establish efficient resistance management tactics before implementing malaria control programmes in Guangxi region.

## References

[1] Enayati A, Hemingway J (2010). Malaria management: past, present, and future. Annu Rev Entomol.

[2] Dai Y, Huang X, Cheng P, Liu L, Wang H, Wang H (2015). Development of insecticide resistance in malaria vector *Anopheles sinensis* populations from Shandong province in China. Malar J..

[3] Fournier D (2005). Mutations of acetylcholinesterase which confer insecticide resistance in insect populations. Chem Biol Interact.

[4] Weill M, Malcolm C, Chandre F, Mogensen K, Berthomieu A, Marquine M (2004). The unique mutation in *ace*-*1* giving high insecticide resistance is easily detectable in mosquito vectors. Insect Mol Biol.

[5] Weill M, Lutfalla G, Mogensen K, Chandre F, Berthomieu A, Berticat C (2003). Insecticide resistance in mosquito vectors. Nature.

[6] Chang X, Zhong D, Fang Q, Hartsel J, Zhou G, Shi L (2014). Multiple resistances and complex mechanisms of *Anopheles sinensis* mosquito: a major obstacle to mosquito-borne diseases control and elimination in China. PLoS Negl Trop Dis.

[7] Djogbenou L, Akogbeto M, Chandre F (2008). Presence of insensitive acetylcholinesterase in wild populations of *Culex pipiens quinquefasciatus* from Benin. Acta Trop.

[8] Liebman KA, Pinto J, Valle J, Palomino M, Vizcaino L, Brogdon W (2015). Novel mutations on the *ace*-*1* gene of the malaria vector *Anopheles albimanus* provide evidence for balancing selection in an area of high insecticide resistance in Peru. Malar J.

[9] Essandoh J, Yawson AE, Weetman D (2013). Acetylcholinesterase (*Ace*-*1*) target site mutation 119S is strongly diagnostic of carbamate and organophosphate resistance in *Anopheles gambiae s.s.* and *Anopheles coluzzii* across southern Ghana. Malar J.

[10] Weetman D, Mitchell SN, Wilding CS, Birks DP, Yawson AE, Essandoh J (2015). Contemporary evolution of resistance at the major insecticide target site gene *Ace*-*1* by mutation and copy number variation in the malaria mosquito *Anopheles gambiae*. Mol Ecol.

[11] Assogba BS, Djogbénou LS, Saizonou J, Milesi P, Djossou L, Djegbe I (2014). Phenotypic effects of concomitant insensitive acetylcholinesterase (*ace*-*1*^*R*^) and knockdown resistance (*kdr*^*R*^)in *Anopheles gambiae*: a hindrance for insecticide resistance management for malaria vector control. Parasit Vectors.

[12] Muthusamy R, Shivakumar MS (2015). Susceptibility status of *Aedes aegypti* (L.) (Diptera: Culicidae) to temephos from three districts of Tamil Nadu, India. J Vector Borne Dis.

[13] Low VL, Chen CD, Lim PE, Lee HL, Lim YA, Tan TK (2013). First molecular genotyping of insensitive acetylcholinesterase associated with malathion resistance in *Culex quinquefasciatus* Say populations in Malaysia. Pest Manag Sci.

[14] Baek JH, Kim HW, Lee WJ, Lee SH (2006). Frequency detection of organophosphate resistance allele in *Anopheles sinensis* (Diptera: Culicidae) populations by real-time PCR amplification of specific allele (rtPASA). J Asia-Pacific Entomol.

[15] Qin Q, Li YJ, Zhong DB, Zhou N, Chang XL, Li CY (2014). Insecticide resistance of *Anopheles sinensis* and *An. vagus* in Hainan Island, a malaria-endemic area of China. Parasit Vectors.

[16] Misra BR, Gore M (2015). Malathion resistance status and mutations in acetyl-cholinesterase gene (*Ace*) in Japanese encephalitis and filariasis vectors from endemic area in India. J Med Entomol.

[17] Li JH, Li J, Qin YX, Guo CK, Huang YM, Lin Z (2014). Appraisal of the effect and measures on control malaria for 60 years in Guangxi. J Trop Med.

[18] Lu GY, Zhou SS, Horstick O, Wang X, Liu YL, Müller O (2014). Malaria outbreaks in China (1990–2013): a systematic review. Malar J.

[19] Joshi D, Park MH, Saeung A, Choochote W, Min GS (2010). Multiplex assay to identify Korean vectors of malaria. Mol Ecol Resour.

[20] Rinkevich FD, Zhang L, Hamm RL, Brady SG, Lazzaro BP, Scott JG (2006). Frequencies of the pyrethroid resistance alleles of *Vssc1* and *CYP6D1* in house flies from the eastern United States. Insect Mol Biol.

[21] Edi CVA, Koudou BG, Jones CM, Weetman D, Ranson H (2012). Multiple-insecticide resistance in *Anopheles gambiae* mosquitoes, Southern Côte d’Ivoire.. Emerg Infect Dis.

[22] Djogbenou L, Weill M, Hougard JM, Raymond M, Akogbeto M, Chandre F (2007). Characterization of insensitive acetylcholinesterase (ace-1R) in *Anopheles gambiae* (Diptera: Culicidae): Resistance levels and dominance. J Med Entomol.

[23] Alout H, Djogbenou L, Berticat C, Chandre F, Weill M (2008). Comparison of *Anopheles gambiae* and *Culex pipiens* acetycholinesterase 1 biochemical properties. Comp Biochem Physiol B: Biochem Mol Biol.

[24] Djogbenou L, Dabire R, Diabate A, Kengne P, Akogbeto M, Hougard JM (2008). Identification and geographic distribution of the ACE-1(R) mutation in the malaria vector *Anopheles gambiae* in south-western Burkina Faso, West Africa. Am J Trop Med Hyg.

[25] Tan WL, Wang ZM, Li CX, Chu HL, Xu Y, Dong YD (2012). First report on co-occurrence knockdown resistance mutations and susceptibility to beta-cypermethrin in *Anopheles sinensis* from Jiangsu Province, China. PLoS One.

